# Temporal power of a cycling sprinter: experiments and effective time theory

**DOI:** 10.1098/rspb.2024.2649

**Published:** 2025-07-09

**Authors:** Christophe Clanet, Iris Sachet, Sylvain Dorel, Caroline Cohen, Baptiste Morel, Maximilien Bowen, Pierre Samozino, Emmanuel Brunet

**Affiliations:** ^1^ LadHyX, CNRS–Ecole Polytechnique, Institut Polytechnique de Paris, Palaiseau 91120, France; ^2^ Fédération Française de Cyclisme, Montigny le Bretoneux, France; ^3^ Nantes Université, Movement–Interactions–Performance, MIP, UR 4334, Nantes F-44000, France; ^4^ University of Savoie Mont Blanc, Interuniversity Laboratory of Human Movement Sciences, EA 7424, Chambéry F-73000, France

**Keywords:** force-velocity-endurance, track cycling, theoretical model for fatigue

## Abstract

Using data recorded on ergocycle and *in situ* at the National Velodrome of Saint-Quentin-en-Yvelines, either during training sessions or during the 2022 World Championships, we first carry out an experimental study of the decay over time of the power produced by a sprinter. We then develop a theoretical model for the power output based on the definition of an effective time to account for fatigue. Comparisons between the predictions of the model and the experimental data show a good agreement in all the conditions tested, both on ergocycle and in the velodrome.

## 1. Introduction

For track cycling, studies began in 1894 with the seminal work of Carlo Bourlet [1–3], which addressed both the motion of a bike and the geometry of velodromes. A review of the characteristics of track cycling was later published by Craig & Norton in 2001 [4], which presented the different events and the characteristics of athletes.

**Figure 1.**
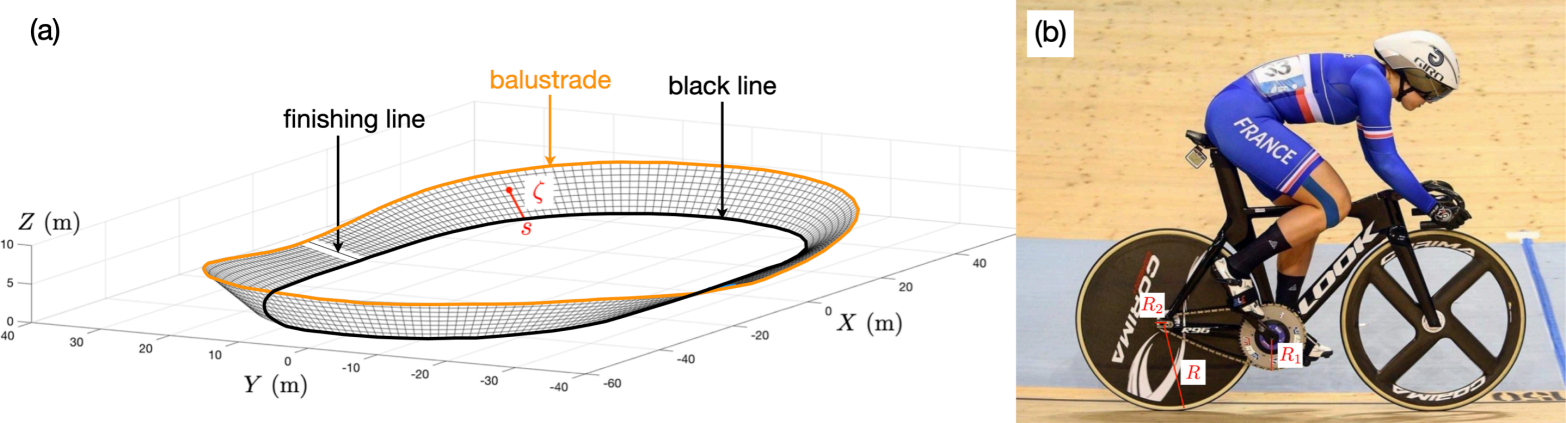
(a) 3D view of the National Velodrome of Saint-Quentin-en-Yvelines, (b) presentation of a track cyclist and track bike and definition of the gear ratio $G=R_{1}/R_{2}$ where $R_{1}$ is the size of the chainring and $R_{2}$ the size of the sprocket. The length *R* is the radius of the wheel.

**Figure 2.**
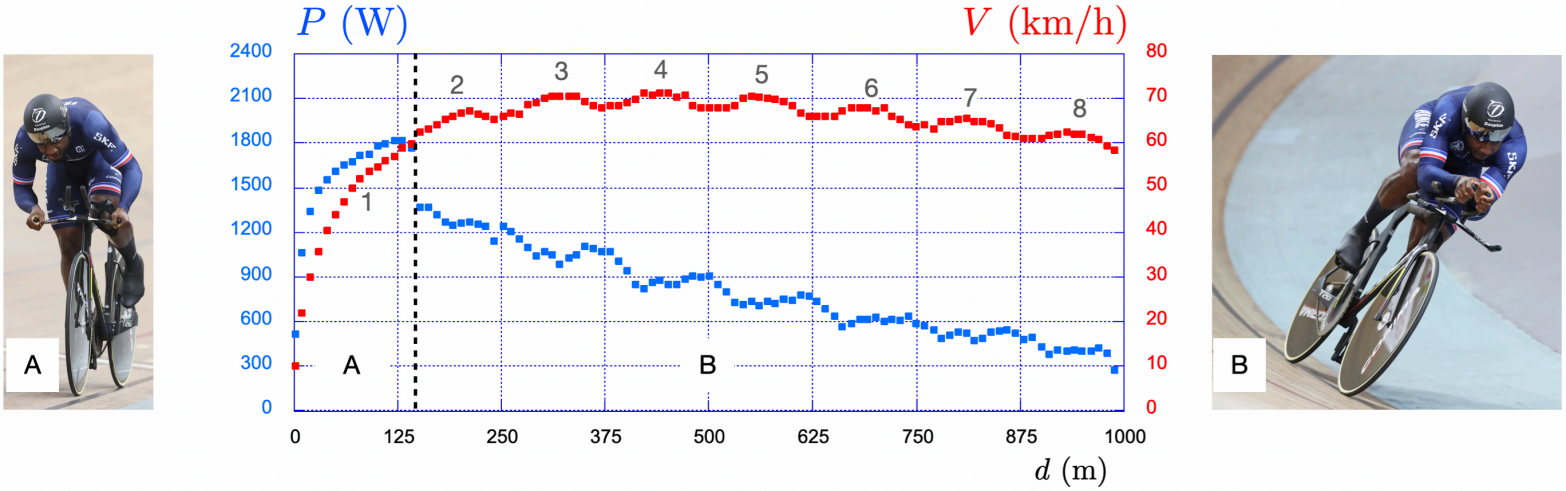
Elite male sprinter in the kilometre time trial at the 2022 World Championships held at the National Velodrome of Saint-Quentin-en-Yvelines. The average power per pedalling cycle is presented in blue and reads on the left vertical scale while the average velocity per pedalling cycle is in red and reads on the right vertical scale. The gear ratio is G=62/13. The dotted vertical black line represents the transition between the initial stand position (A) and the final seated position (B).

A key difference between road and track cycling is the shape of the track, which differs in many ways from a road, starting with its banking up to 45° in turns [5,6]. A three-dimensional view of the National Velodrome of Saint-Quentin-en-Yvelines (SQY) is shown in figure 1a. To locate a cyclist on the velodrome, we use the position along the black line, s, and the distance, ζ, from this line. The width of a velodrome is constant and is 8 m at SQY (ζ=8 m at the balustrade). The length of a lap at the black line (ζ=0) is 250 m, a standard imposed by the International Cycling Union (UCI) for Olympic velodromes since 1992. Beyond the geometry of the track, a fundamental distinction in track cycling lies in the use of a fixed-gear bicycle, which lacks both brakes and a derailleur. The fixed-gear ratio $G=R_{1}/R_{2}$ is defined in figure 1b where $R_{1}$ is the size of the chainring and $R_{2}$ the size of the sprocket. The velocity of the bike V is then directly related to the pedalling rate $\dot{\theta }$ by the relationship $V=RG\dot{\theta }$, where R is the radius of the wheel. Typically R=0.334 m so that with $\dot{\theta }=12\text{ rad/s}$ (114 RPM) and G=62/13 one finds $V=19.1ms^{-1}$ (69 km/h). These values correspond to the data presented in figure 2. This fixed relationship between pedalling rate and velocity directly impacts the performance and the determination of the optimal gear ratio for a given athlete on a given race stands as one of the major problem in track cycling [7,8].

In figure 2, we present the data recorded during the 2022 World Championships held at SQY, using a dedicated instrumented crank developed with the company Phyling, which allows the recording at 200 Hz of both the pedalling rate with a gyroscope and the torque with strain gauges (§2b(i)). These data correspond to the kilometre time trial of the elite sprinter involved in the present study where he achieved 59.568 s using a gear ratio G=62/13. Since the radius of a wheel is R=0.334 m, one deduces that the distance covered during a single pedalling revolution is 2πRG=10.01 m. The kilometre is thus covered with 100 pedal turns.

The mean power per pedalling cycle is defined as $P(t)=1/T\int_{t}^{t+T}P_{i}(t^{\prime })dt^{\prime }$ where $P_{i}(t)$ is the instantaneous power at time t and T the duration of the pedalling cycle (from t to t+T). It is shown with blue squares and reads on the left axis, while the speed in red squares reads on the right axis. After the standing start, the sprinter remains in the stand position (A) during 140 m and then sits and remains seated until the end of the kilometre (B). The dotted vertical black line represents the stand-seat transition. The mean power per pedalling cycle initially increases up to 1800 W in a non-linear way, drops to 1300 W at the stand-sit transition and then continuously decreases down to 400 W at the end of the race. Concerning the speed, it increases strongly during the stand phase, reaches a plateau around 70 km/h after 300 m, and then slightly decreases to reach 60 km/h when completing the kilometre.

Such a relationship between power output and speed has already been reported in the literature for different distances of races [9,10]. Qualitatively, the patterns of power and speed are similar even if obtained with different elite athletes over different distances: the power always peaks in the stand phase, drops at the stand-sit transition and then continues to decay in the seated position. Concerning the speed, it primarily increases during the stand phase, reaches a maximum in the seated phase and slowly decreases after 500 m.

**Figure 3.**
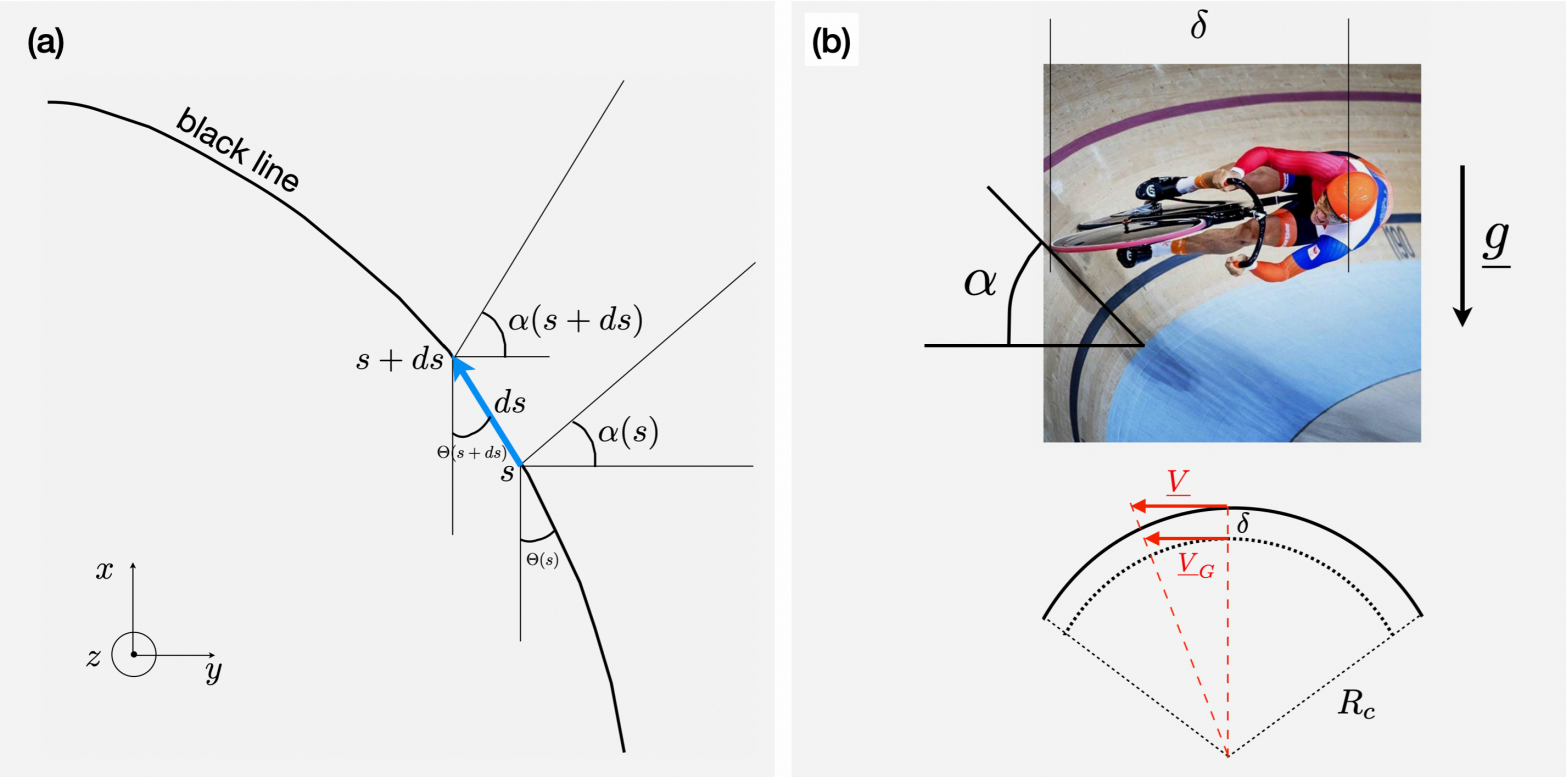
(a) Presentation of the banking angle, α, and of the azimuth angle, Θ. (b) Definition of the distance δ and illustration of the difference between the velocity of the centre of mass, $V_{G}$, and the velocity of the bike, V.

In all cases, we also notice some oscillations in the speed profile which have been attributed to the inclination of the sprinter in turns (see figure 3b) [11–13]. As the track has 2 turns in 250 m, the kilometre is covered in 4 laps, i.e. 8 turns inducing the 8 oscillations observed in figure 2.

The theoretical relationship between the athlete’s power output and the resulting speed on a track has been studied by several authors [11–13]. These studies have shown that if the average power per pedalling cycle is known, the resulting speed can be deduced with high accuracy using two equations (§3a).

However, the average power per pedalling cycle is only known at the end of the race, and not beforehand, which is essential if one wants to determine in advance the optimal gear or the optimal pacing strategy.

The goal of the present study is to establish a closure relationship that can predict the time evolution of the power per pedalling cycle of a sprinter, taking fatigue into account.

## 2. Experimental results

Our case study focuses on data collected from an elite male sprinter (25 years old, height $L_{c}=1.85m$, weight $M_{c}=92\mathrm{kg}$), specializing in sprint events including match sprint, team sprint, keirin and kilometre. We first present his biomechanical characteristics as measured on an ergocycle and subsequently highlight some of his track performances, recorded during both training sessions and competitions.

### (a) Experimental data on ergocycle

#### (i) Torque and power without fatigue

At the scale of multi-joint movements, the force–velocity relationship is well described by a linear regression [14–16]. For cycling, this linearity is observed between the torque, Γ, exerted on the cranks and the pedalling rate, $\dot{\theta }$ [17–21].

**Figure 4.**
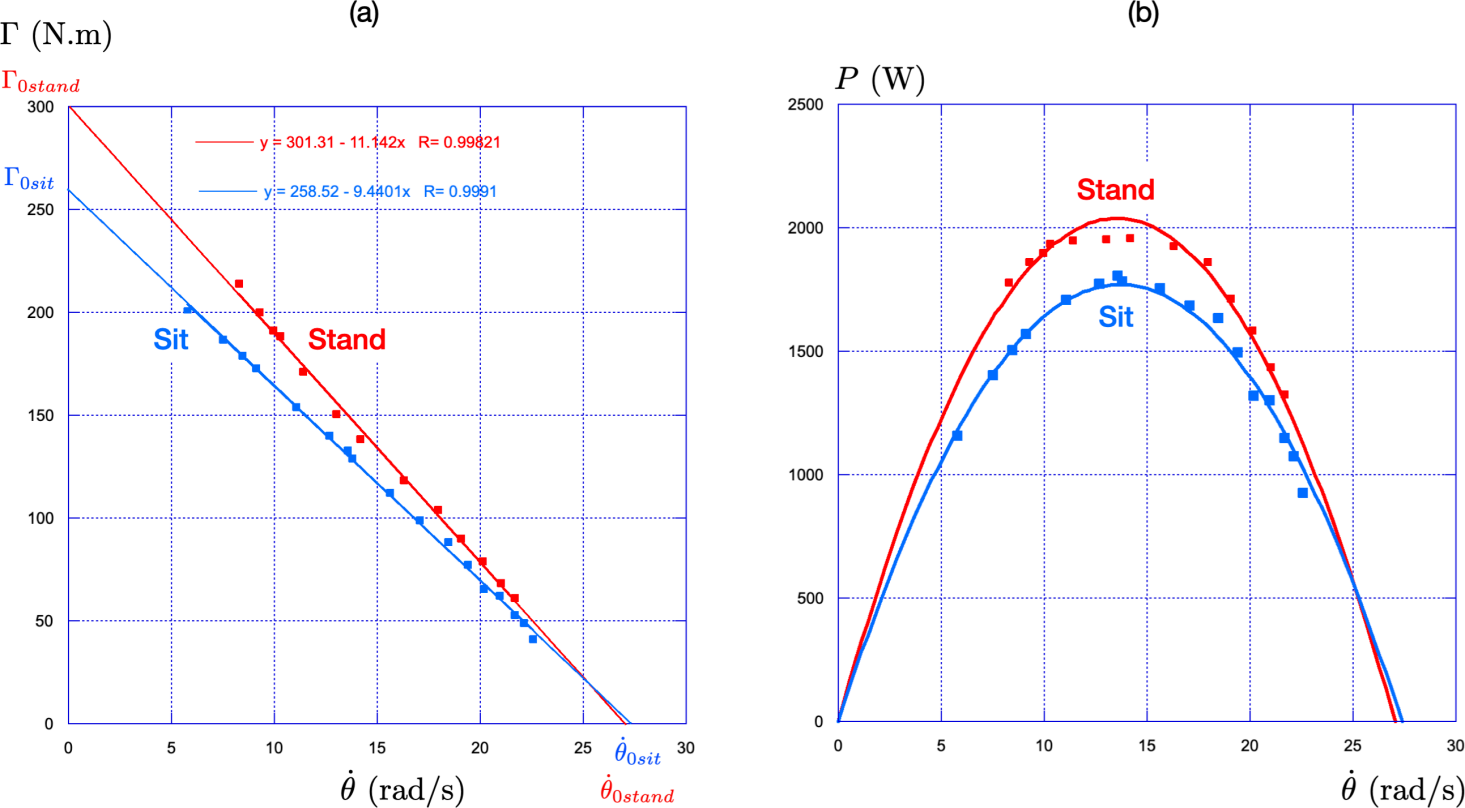
(a) Evolution of the maximal torque, Γ, with the pedalling rate, $\dot{\theta }$, for the stand position (in red) and for the seated position (in blue) (b) Associated evolution of the maximal power $P=\Gamma .\dot{\theta }$ with the pedalling rate. The convention for the colour is the same as in (a).

Using a Lode Excalibur ergocycle equipped with force sensors, the athlete performed three sprints of 3–5 s either in stand or seated position with a 5 min recovery period between each trial. The ergometric bicycle was equipped with force sensors at each crank, allowing the measurement of the total torque. The crank angle was measured from TTL signals delivered every 2° of rotation.^1^ The torque, angular velocity and power output were averaged over each pedal cycle following the procedure detailed in [22]. The resulting evolution of the maximal torque, Γ, with the pedalling rate, $\dot{\theta }$, is presented in figure 4a for the two positions, stand in red and seated in blue.

In both cases, we observe a linear decrease of the maximal torque with the pedalling rate: $\Gamma =\Gamma_{0stand}\left(1-\dot{\theta }/{\dot{\theta }}_{0stand}\right)$ for the stand position, and $\Gamma =\Gamma_{0sit}\left(1-\dot{\theta }/{\dot{\theta }}_{0sit}\right)$ for the seated position. $\Gamma_{0sit}$ and $\Gamma_{0stand}$, respectively, represent the maximal values of the torque in sit and stand positions. Similarly, ${\dot{\theta }}_{0sit}$ and ${\dot{\theta }}_{0stand}$ stand for the maximal value of the pedalling rate in sit and stand positions. These four parameters are shown in figure 4a.

We observe that the maximal pedalling rate is similar in both positions (${\dot{\theta }}_{0sit}\approx {\dot{\theta }}_{0stand}\approx 27rad/s\left(258\mathrm{RPM}\right)$), but the maximal torque, obtained at zero pedalling rate, is larger in the stand position, $\Gamma_{0stand}=301.3N.m$, than in the seated position, $\Gamma_{0sit}=258.5N.m$. This justifies the initial stand position in all standing-start races.

From these data, we deduce the associated power as $P=\Gamma \;\dot{\theta }$. In this equation, the power, P, is in W, the torque, Γ, in N.m and the pedalling rate, $\dot{\theta }$, in rad/s. The conversion in RPM is obtained through the relationship ${\dot{\theta }}_{RPM}=9.549\;{\dot{\theta }}_{rad/s}$. The resulting parabolic behaviour of the power is shown in figure 4b, highlighting two key characteristics:

— An optimal pedalling rate exists at which the power is maximal. In this case, it is approximately 13.5 rad/s (130 RPM).
— The maximal power is higher in the stand position, with a difference of 13% compared to the seated position (2020 W in the stand position versus 1800 W in the seated position).

These observations are consistent with previous data reported for track cyclists [20].

#### (ii) Torque and power with fatigue

The literature indicates that the power generated during maximal efforts decreases over time from a maximum value to a critical value. The maximum power can be sustained for only a few seconds (3–5 s), whereas the critical power can be maintained for several minutes [23–25]. The decline in power during prolonged sprint exercises has been shown to depend on the pedalling rate [9,17,18].

For our elite sprinter, the evolution of the maximal power during sustained maximal efforts of 60 s is measured using two isokinetic sprints performed on a cycle ergometer, one at 130 RPM ($\dot{\theta }=13.6\text{ rad/s}$) and one at 91 RPM ($\dot{\theta }=9.53\text{ rad/s}$). In isokinetic mode, the pedalling rate is controlled and maintained fixed throughout the sprint by the servomotor on the Lode Excalibur ergometer via the adjustment of a variable resistance.

The choice of these pedalling rates is based on the data of the fatigue-free characteristics reported in figure 4, which indicate an optimal pedalling rate of 130 RPM. Since one expects this optimal rate to decrease with time due to fatigue, the second pedalling rate is chosen as 70% of the initial optimum, resulting in 91 RPM. These values were selected to obtain clear differences in the power-time curve, particularly in its alteration over the 60 s.

**Figure 5.**
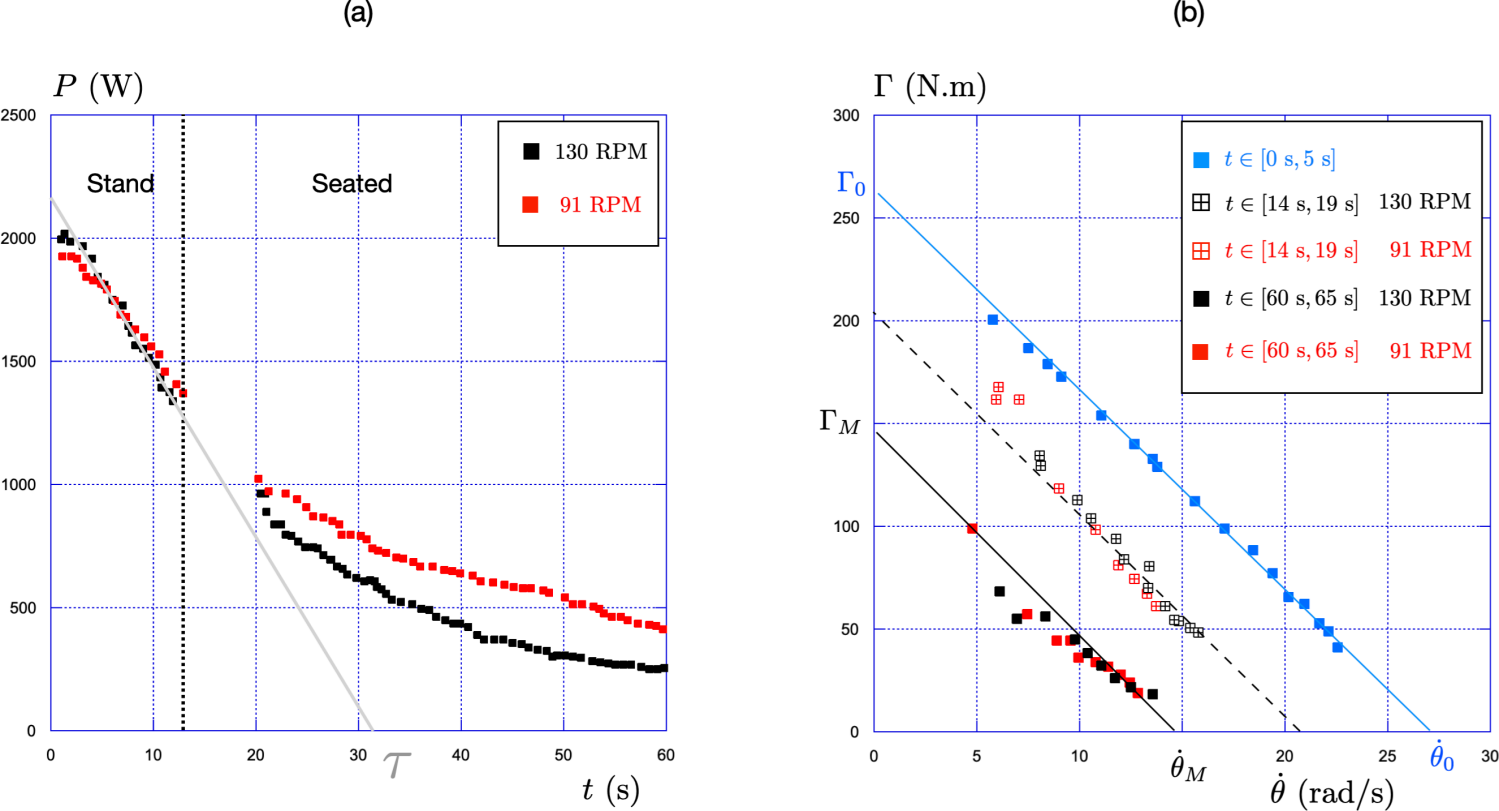
(a) Evolution of the maximal power, $P=\Gamma .\dot{\theta }$, with time for two isokinetic sprints (black square = 130 RPM, red square = 91 RPM) (b) Evolution of the maximal torque, Γ, with the pedalling rate, $\dot{\theta }$, for the fatigue-free seated condition (blue squares) and the fatigue condition at 14 s (red and black hollow crossed squares) and 60 s (red and black filled squares) for two pedalling rates (130 RPM in black and 91 RPM in red). The solid and dotted lines are guides for eyes parallel to each other.

The results for the maximal power per pedalling cycle are presented in figure 5a. The first 13 s are performed in the standing position, while the last 40 s of the 60 s all-out sprint are done seated. The power decreases with a characteristic time τ of the order of 30 s. The rate of decrease depends on the pedalling rate: it is more rapid at 130 RPM than at 91 RPM.

In order to assess the torque-cadence, $\Gamma (\dot{\theta })$, relationships in the fatigued state after the first 14 s and at the end of the 60 s sprint, we used the unique sprint inertial-loaded method [26]: We measure the torque-pedalling rate relationship over two 5 s periods: from 14 to 19 s (referred to as $t_{14}$) which explains the lack of data in this time interval in figure 5a and from 60 to 65 s (referred to as $t_{60}$).

The results are presented in figure 5b and compared to the results obtained without fatigue in the seated position (same results as in figure 4, presented here in blue and referred to as $t_{0}$. So in figure 5b, $\Gamma_{0}=\Gamma_{0_{sit}}$ and ${\dot{\theta }}_{0}={\dot{\theta }}_{0_{sit}}$). The results with fatigue are shown in black for the 130 RPM and in red for the 91 RPM.

We first observe that for the three cases $t_{0}$, $t_{14}$, and $t_{60}$, the maximal torque still decreases linearly with the pedalling rate, following the relation $\Gamma =\Gamma_{M}\left(1-\dot{\theta }/{\dot{\theta }}_{M}\right)$. For $t_{0}$, $\Gamma_{M}=\Gamma_{0}$ and ${\dot{\theta }}_{M}={\dot{\theta }}_{0}$.

The second observation, highlighted by the solid and dashed parallel lines, is that the entire curve appears to shift to the left, while maintaining a constant slope: $\Gamma_{M}=\Gamma_{0}/K$ and ${\dot{\theta }}_{M}={\dot{\theta }}_{0}/K$, with the constant K increasing with the duration of the effort (K=1 for $t_{0}$, K=1.3 for $t_{14}$ and K=2 for $t_{60}$).

All these observations provide experimental evidence that the maximal pedalling rate decreases with fatigue, which is consistent with recent findings in the literature [9,27].

### (b) Experimental data on track

#### (i) A dedicated sensor for on-field measurements

To obtain on-field measurements both during training sessions and in competitions, we have developed a high-frequency (200Hz) force and speed sensor in collaboration with the company Phyling and obtained UCI approval in 2020 for its used in all competitions.

**Figure 6.**
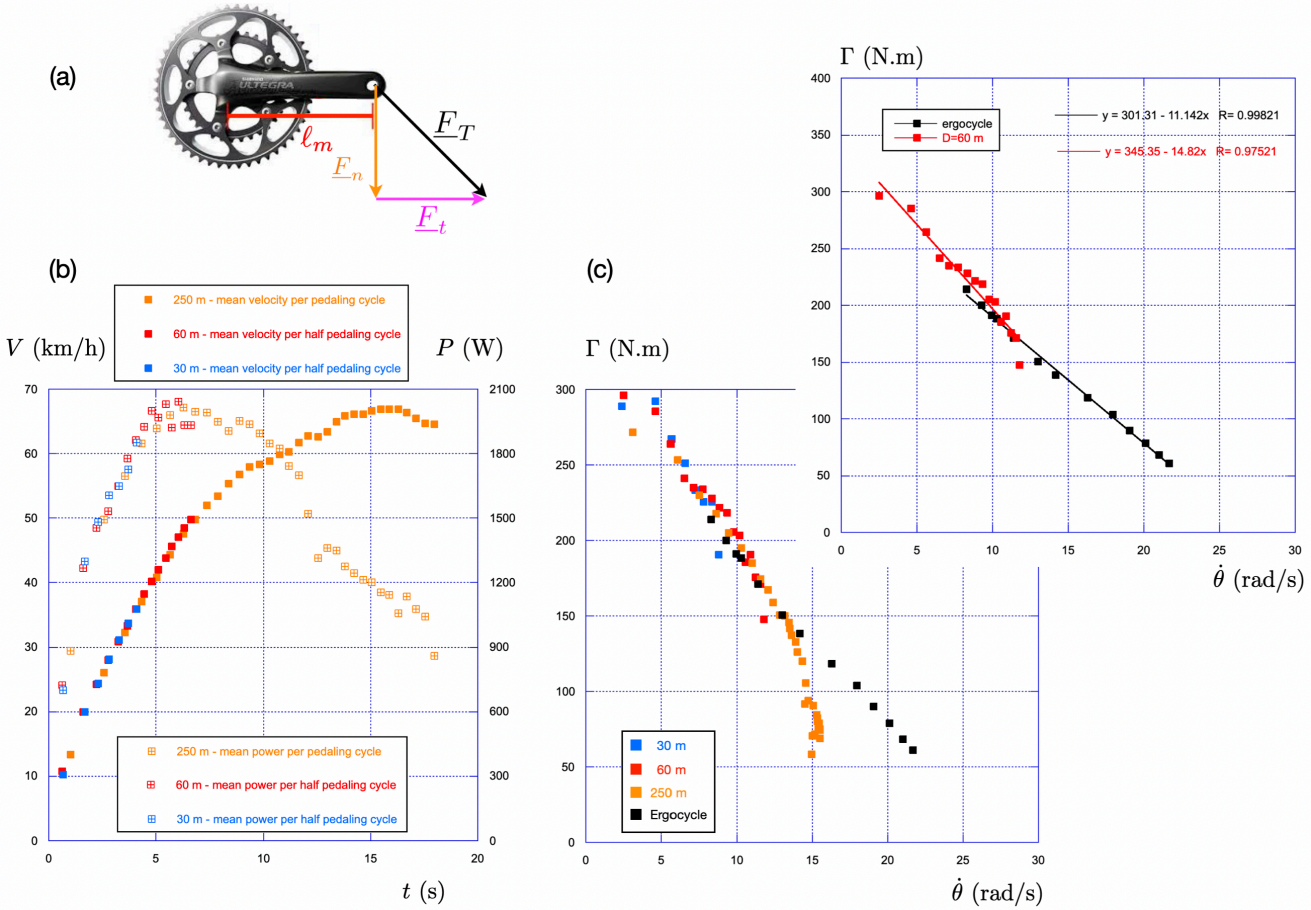
(a) Description of the pedalling crank with the decomposition of the applied force in normal and tangential components. (b) Time evolution of the velocity (full symbol) and of the power (hollow crossed symbol) for three standing starts of different distances: 30 m (blue), 60 m (red) and 250 m (orange). The gear ratio for the three distances is the same: G=61/17. (c) Associated torque-pedalling rate relationship using the same convention of colour. In black, we replot the data of figure 4 obtained on the Lode ergocycle in the stand position.

The total force, $\underline{F}{\;}_{T}$, exerted by the cyclist includes a propulsive part normal to the crank, $\underline{F}{\;}_{n}$, and a non-propulsive part tangent to the crank, $\underline{F}{\;}_{t}$ (figure 6a). The normal force $\underline{F}{\;}_{n}$ is measured with strain gauges on each crank: $\underline{F}{\;}_{nl}$ for the left leg and $\underline{F}{\;}_{nr}$ for the right leg. Using the crank length $\ell_{m}$, one deduces the pedalling torque $\Gamma =\left(F_{nl}+F_{nr}\right)\;\ell_{m}$.

The instantaneous pedalling rate $\dot{\theta }$ is measured at high frequency with a gyroscope, while the average pedalling rate is measured using a setup consisting of a magnet fixed to the bike frame and a magnetometer installed on the crank.

The instantaneous power $P_{i}=\left(F_{nl}+F_{nr}\right)\;\ell_{m}\;\dot{\theta }$ is calculated from these measurements and then averaged over the pedalling cycle to obtain the mean power P discussed in the present paper.

#### (ii) Example of training session

During a training session, the elite sprinter used a gear ratio G=61/17 to perform three standing starts of different distances: 30, 60 and 250m. The air density was measured that day to be $\rho =1.157kg/m^{3}$. For 30 and 60m, the sprinter remains in the stand position from start to finish. For 250m, he sits down after 13 s, i.e. after having covered a distance of 140m.

The results are presented in figure 6: the time evolution of the speed and power are shown in (b) for the three standing starts. We observe that the evolutions of the speed and power do not depend on the distance of the race.

The associated torque-pedalling rate relationships are shown in figure 6c for the three-time trials. Since the sprinter remains in the stand position during the first 140m, we also plot in black the data obtained on the Lode ergocycle in the standing position for short sprints without fatigue (same data as those presented in figure 4.

We observe that the data for the 30m (final time 4.3 s) and 60m (final time 6.6 s) are close to the data obtained on the Lode ergocycle for short sprints in the standing position without fatigue. The inset in figure 6c reveals that the maximal torque on track is 15% larger than that measured on the ergocycle ($\Gamma_{0stand}(\text{track})=345N.m$ and $\Gamma_{0stand}(\text{ergocycle})=301N.m$). This difference depends on the athlete and must be taken into account when comparing ergocycle and on-track experiments.

For the 250m race (final time 18 s), the data are also close to those obtained with the ergocycle up to 13 s, after which the torque decreases in a strongly nonlinear way due to fatigue and to the stand-sit transition occurring around 13 s.

#### (iii) Impact of the gear ratio, G

**Figure 7.**
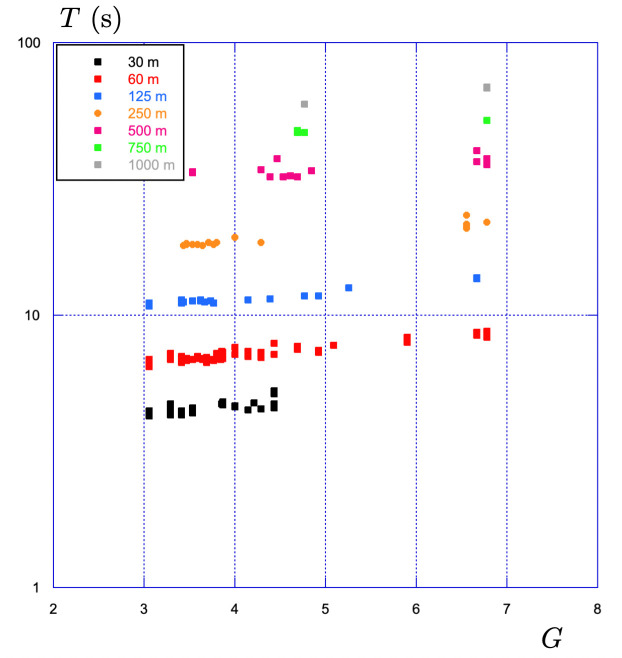
Evolution of the time of the race T with the gear ratio G for different distance of races ranging from 30 m to 1000 m performed during training sessions.

All along his training sessions, the elite sprinter has used many different gear ratios over various distances to develop his strength and endurance. In figure 7, we show the evolution of the final time of the race T as a function of the gear ratio G for races of different distances ranging from 30 to 1000m. The general observation is that the race time increases as the gear ratio increases from 3 to 7. With a gear of 3.5 (e.g. 49/14), the distance covered per pedal revolution is 3.5 times the perimeter, i.e. 7.34m. With a gear of 6.78 (61/9), the distance becomes 14.2m.

For the 250 m race (points in orange), the race time is 18 s using a gear ratio G=3.5, and it becomes 21.7 s with a gear ratio G=6.78, which corresponds to a 20% increase.

## 3. Theoretical model

### (a) From power to velocity

The relationship between the athlete’s power output per pedalling cycle, P, and the resulting speed of the bike, V, in a velodrome has been studied by several authors [11–13]. It can be reduced to two equations: one for the velocity of the centre of mass of the system (bike +cyclist), $V_{G}$, derived on energy conservation, and one for the speed of the bike V derived on kinematics and function of the geometry of the velodrome:


(3.1a)ddt(12MVG2)=ηP−12ρSCDVG3−CRRMgV[cos⁡α+(VG2/gRc)sin⁡α](3.1b)V=VG1−δ/Rc with δ=LG⋅(VG2/gRc)/1+(VG2/gRc)2.

This set of equations holds for races along the black line for which the potential energy remains constant. The banking angle α and the azimuth angle Θ are presented in figure 3a: The banking angle α measures the inclination of the track with respect to the horizontal. At SQY, it varies from 14° in straight lines to 44° in turns. The azimuth Θ measures the angle between the local tangent to the black line and a fixed direction, here the direction [Ox). The curvature $\Theta^{\prime }=d\Theta /ds=1/R_{c}$ is illustrated in figure 3b. These geometrical characteristics, α(s) and Θ(s), depend on the velodrome [5,6].

Back to equation (3.1a), the mass M=100kg is the sum of the mass of the bike $M_{b}=8\mathrm{kg}$ and the mass of the athlete $M_{c}=92\mathrm{kg}$.

The first term on the right-hand side of equation (3.1a) is the effective power ηP dedicated to the forward motion of the bike. It is composed of the pedalling power P and the global transmission efficiency η, which accounts for the mechanical efficiency, steering efficiency and motion efficiency of the rider. For the entire study, we use η=0.91 [28].

The remaining two terms on the right-hand side of equation (3.1a) respectively represent the power dissipated by aerodynamic resistance, $\frac{1}{2}\rho SC_{D}V_{G}^{3}$, and by rolling friction, $C_{RR}MgV\left(\cos\alpha +\frac{V_{G}^{2}}{gR_{c}}\sin\alpha \right)$.

The drag area, $SC_{D}$, is measured in a wind tunnel and depends on the cyclist’s position on the bike: for the elite sprinter involved in the study, we measure $SC_{D}=0.27\;m^{2}$ in the standing position and $SC_{D}=0.21\;m^{2}$ in the seated position.

Regarding the rolling friction coefficient $C_{RR}$, it was measured on a drum bench [29]. For Vittoria Pista tyres inflated to 12 bars, we find $C_{RR}=0.0025$ with a weak dependency on velocity.

The length $L_{G}$, which appears in equation (3.1b), is related to the cyclist’s size $L_{c}$. Typically, $L_{G}=0.7L_{c}$ [30], leading to $L_{G}=1.30m$ for the elite sprinter ($L_{c}=1.85m$).

If the athlete’s power output, P, is known, the set of equations (3.1a) and (3.1b) can be integrated numerically using the initial condition for standing starts ($V_{G}(t=0)=0$) to obtain an accurate evaluation of the time evolution of the bike velocity, V(t).

### (b) From velocity to power: the effective time theory

The problem is now to find a model for the athlete’s power output, P, able to account for the different experimental behaviours presented in §2.

The athlete’s power output is the product of the pedalling torque Γ and the pedalling rate $\dot{\theta }$: $P=\Gamma \dot{\theta }$. As shown in figure 5b, the maximal pedalling torque Γ is a linearly decreasing function of the pedalling rate at all times, $\Gamma =\Gamma_{M}\left(1-\dot{\theta }/{\dot{\theta }}_{M}\right)$, where the two characteristics $\Gamma_{M}$ and ${\dot{\theta }}_{M}$ depend on fatigue.

Returning to the pedalling power, one deduces from the above relations its dependency on the pedalling rate: $P=4P_{M}\left(1-\dot{\theta }/{\dot{\theta }}_{M}\right)\dot{\theta }/{\dot{\theta }}_{M}$, where $P_{M}=\Gamma_{M}{\dot{\theta }}_{M}/4$ is the maximal value of the power. This parabolic behaviour of the power P with the pedalling rate $\dot{\theta }$ has been observed and discussed in §2a.

The challenge is to describe the impact of fatigue on the pedalling power, i.e., to model the time evolution of the two characteristics $\Gamma_{M}$ and ${\dot{\theta }}_{M}$, knowing that $\Gamma_{M}(t=0)=\Gamma_{0}$ and ${\dot{\theta }}_{M}(t=0)={\dot{\theta }}_{0}$.

The fatigue, i.e. a decrease in maximum capacities, is not a function of the actual time but depends on the energy spent above the critical power $P_{C}$ [31]. The critical power $P_{C}$ corresponds to a threshold under which a physiological stable state is reached [32] with only very limited fatigue development [33]. We thus assume no fatigue at $P=P_{C}$. The energy spent during the time interval dt is P⋅dt which can be decomposed as $P_{C}\cdot dt+(P-P_{C})\cdot dt$. Only the second part induces fatigue. Under maximal conditions ($P=P_{M}$), this energy $(P-P_{C})\cdot dt$ can be spent in the effective time interval $dt_{eff}$ such that $(P_{M}-P_{C})\cdot dt_{eff}=(P-P_{C})\cdot dt$. This energy balance leads to the evolution law of the effective time:


(3.2) $$\frac{dt_{eff}}{dt}=\frac{P-P_{C}}{P_{M}-P_{C}}.$$

This differential equation for the effective time states that $dt_{eff}/dt=0$ for $P=P_{C}$, so the effective time remains constant at the critical power. Equation (3.2) also implies that $dt_{eff}=dt$ at maximal power when $P=P_{M}$. Finally, equation (3.2) suggests that $dt_{eff}/dt<0$ occurs when the cyclist decides to ride with a power below the critical power, $P<P_{C}$. The physical meaning of this negative value of $dt_{eff}$ (dt is always positive) is that the cyclist is in a recovery phase. This recovery phase is bounded by the condition $t_{eff}\geq 0$ at all times.^2^ The effective time is thus defined by equation (3.3a):


(3.3a)J(t)=∫0t(P−PCPM−PC)dt′(3.3b)teff(t)={0if J(t)≤0J(t)if J(t)>0.

Once the effective time $t_{eff}$ is defined, one must discuss its impact on the evolution of the two characteristics $\Gamma_{M}$ and ${\dot{\theta }}_{M}$. We choose a hyperbolic damping $1/(1+t_{eff}/\tau )$ so that when $t_{eff}=\tau$, the quantity is halved. The effective time theory for the evolution of the pedalling power in races can thus be summarized mathematically by the following system:


(3.4a)P=Γ⋅θ˙(3.4b)Γ=ΓM(1−θ˙θ˙M)(3.4c)ΓM=Γ01+teff/τ(3.4d)θ˙M=θ˙01+teff/τ(3.4e)PM=ΓM⋅θ˙M4.

The critical power involved in the definition of the effective time (3.3) is not a constant but depends on the pedalling rate: for the elite sprinter involved in the study, his critical power is $P_{C}=270W$ at $\dot{\theta }=90\mathrm{RPM}\left(=9.42rad/s\right)$ and decreases to $P_{C}=0$ at $\dot{\theta }=130\mathrm{RPM}\left(=13.6rad/s\right)$. The model we use for the critical power is similar to the one used for the pedalling power:


(3.5a)ΓC=ΓCM(1−θ˙θ˙CM)(3.5b)PC={0if ΓC≤0ΓC.θ˙if ΓC>0.

Using $\Gamma_{CM}=93.15\;N.m$ and ${\dot{\theta }}_{CM}=13.6\;rad/s$ we find $P_{C}=270\;W$ for $\dot{\theta }=9.42\;rad/s$ and $P_{C}=0$ for $\dot{\theta }=130\;\mathrm{RPM}\left(=13.6\;rad/s\right)$.

All the constants that appear in the sets of equations (3.3)–(3.5) can be estimated using the data obtained either on the Lode ergocycle or on the track.

## 4. Comparing theory and experiment

### (a) Comparison with the data obtained on the Lode with fatigue

To test the effective time theory, we first compare its predictions with the data obtained on the Lode with fatigue, as presented in figure 5. The system equations (3.3)–(3.5) is integrated for the two pedalling rates $\dot{\theta }=130\mathrm{RPM}=13.61rad/s$ and $\dot{\theta }=91\mathrm{RPM}=9.53rad/s$ using the standing start values of $\Gamma_{0stand}$ and ${\dot{\theta }}_{0stand}$ for the first 13 s ($\Gamma_{0stand}=310N.m$, ${\dot{\theta }}_{0stand}=27rad/s$), and their seated values from 13 to 60 s ($\Gamma_{0sit}=258N.m$, ${\dot{\theta }}_{0sit}=27rad/s$). The characteristic time for the decay of the power is kept constant for all cases, τ=30s.

**Figure 8.**
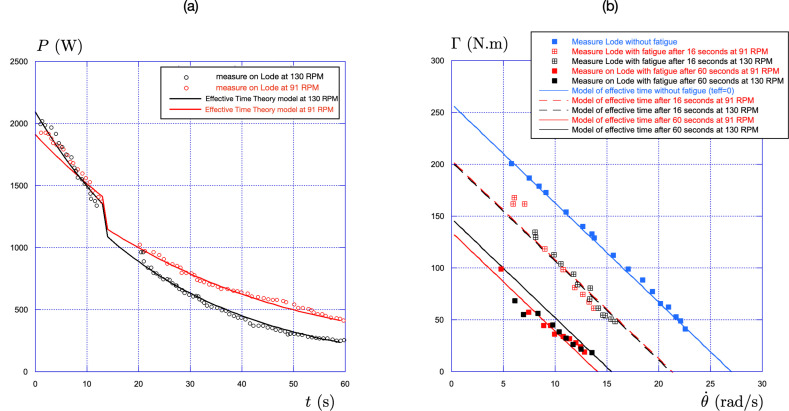
(a) Comparison between the measurement (hollow circles) and the effective time theory (solid lines) for the evolution of the maximal power $P=\Gamma .\dot{\theta }$ with time for two isokinetic sprints performed at maximal effort (black = 130 RPM, red = 91 RPM). (b) Comparison between the measurement (squares) and the effective time theory (lines) for the evolution of the maximal torque Γ with the pedalling rate $\dot{\theta }$ for the fatigue-free seated condition (blue) and the fatigue condition at 14 s ($t_{14}$ red and black hollow crossed squares) and at 60 s ($t_{60}$ red and black filled squares) for two pedalling rates (black = 130 RPM, red = 91 RPM).

The time evolution of the power is shown in figure 8a for the two isokinetic efforts. We observe a fair agreement between the experimental data (hollow circles) and the effective time theory (solid lines).

After 14 s and at the end of the 60 s effort, the torque-pedalling rate relationship was measured and compared to the one without fatigue (figure 5b). The same protocol is used with the effective time theory, and the comparison with the measurements is shown in figure 8b. Again, the agreement is fair for the two isokinetic efforts (91 RPM and 130 RPM) and for the two different fatigue times (14 s, $t_{14}$, and 60 s, $t_{60}$).

### (b) Comparison with the data obtained on track

The set of equation (3.4) provides an expression for the pedalling power P:


(4.1) $$P=\left(\frac{\Gamma_{0}}{1+t_{eff}/\tau }-\frac{\Gamma_{0}}{{\dot{\theta }}_{0}}\frac{V}{RG}\right).\frac{V}{RG}.$$

To derive this closure equation from the system (equation (3.4)), we have used the linear relationship between the pedalling rate and the bike speed, $V=RG\dot{\theta }$, where R represents the radius of the wheel and G is the gear ratio.

Since the initial condition is V(t=0)=0, equation (4.1) implies that the initial value of the power is null, which in turn implies that the speed should remain null at all times equation (3.1a). This initial value paradox is solved by changing the differential equation in time (3.1a) to the corresponding differential equation in space, using dt=ds/V:


(4.2) $$\frac{dV_{G}^{2}}{ds}=\frac{2}{M}\left[\eta \frac{\Gamma }{RG}-\frac{1}{2}\rho SC_{D}V_{G}^{2}\left(1-\delta /R_{c}\right)-C_{RR}Mg\left(\cos\alpha +(V_{G}^{2}/gR_{c})\sin\alpha \right)\right].$$

Initially, since $t_{eff}(0)=0$, the torque is maximum, $\Gamma =\Gamma_{0}$ (equation (3.4b) and equation (3.4c)), and equation (4.2) indicates that, in this case, the cyclist accelerates following the law


$${\left(\frac{dV_{G}^{2}}{ds}\right)}_{t=0}=\frac{2\eta \Gamma_{0}}{MRG}.$$

The lower the gear ratio G, the larger the acceleration, which is physically meaningful.

The numerical integration of the systems (3.1), (3.3)–(3.5) using equation (4.2) instead of (3.1a) is performed below for the two different races presented in §2b, namely the 1-lap standing start performed with the gear ratio G=61/17 (figure 6) and the kilometre time trial performed with G=62/13 (figure 2).

#### (i) The 1 lap D=250 m with G=61/17

Three standing start races of 30, 60 and 250m performed at SQY with G=61/17 are presented in figure 6, showing the time evolution of the speed and power in figure 6b and the evolution of the torque with the pedalling rate in figure 6c. Since these data show that there is no influence of the distance on these characteristics, we focus the comparison with the effective time theory on the longer race, which is the 1-lap 250 m.

**Figure 9.**
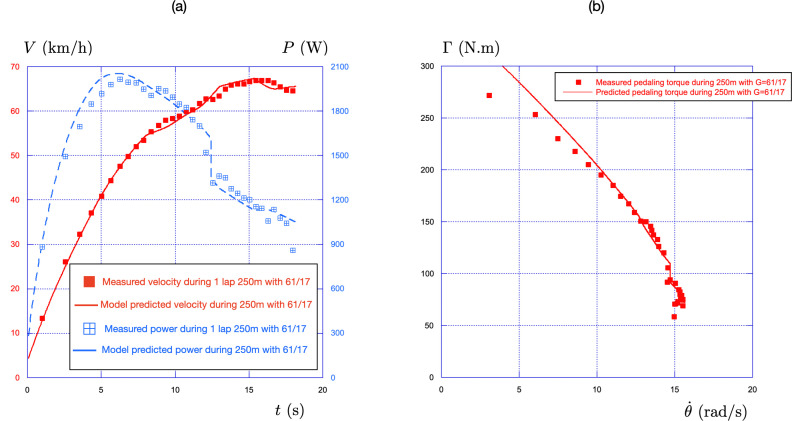
(a) Comparison experiment-theory of the time evolution of the speed and of the power for a 1 lap standing start (250 m) performed with the gear ratio G=61/17. The experiments are shown with squares (filled for speed and hollow crossed for power). The predictions of the effective time theory are shown with lines (solid for the velocity and dashed for the power). (b) Associated torque-pedalling rate relationship using the same convention: hollow square for experimental measures and solid line for the effective time theory.

Due to the location of the stand-sit transition, the first 140 m are integrated with the values of $\Gamma_{0}$ and ${\dot{\theta }}_{0}$ obtained from on-track measurements in the standing position ($\Gamma_{0}=\Gamma_{0_{stand}}=353N.m$, ${\dot{\theta }}_{0}={\dot{\theta }}_{0_{stand}}=27rad/s$). The end of the race, from 140 to 250 m, is performed seated, and the numerical integration uses the corresponding on-track values: $\Gamma_{0}=294N.m$ and ${\dot{\theta }}_{0}=27rad/s$. The results are presented in figure 9: the time evolution of both the power and the velocity in (a) and the torque-pedalling rate relationship in (b).

For the time evolution of the power, the general behaviour—with a peak value during the standing phase, a drop of 300 W during the stand-sit transition, and a decrease in the seated position—is well captured by the effective time theory. The main difference lies in the position of the peak power value, which is found to be 2057 W by the model instead of the 2010 W measured by the sensor. This 2.3% difference serves as a first evaluation of the accuracy of the model. The associated evolution of the velocity (in red) also shows fair agreement, with a slight phase shift observed in turns.

The torque-pedalling rate relationship shown in figure 9b reveals a 14% overshoot for the first pedalling cycle, a 5.5% overshoot for the second, and an agreement below 5% for the rest of the race. The model also provides a fair description of both the stand-sit transition (vertical drop observed at $\dot{\theta }=14.6rad/s$) and of the fatigue in the seated phase.

The main difference between the on-field data and the model is therefore observed in the low pedalling rate regime, that is near the start. It is important to note that a standing start in track cycling is achieved using a starting gate that initially holds the bike in place by clamping the rear wheel. The release of this mechanism is synchronized with the countdown, allowing the sprinter to time their movement for the quickest possible exit. From our perspective, the deviations observed at low cadences are associated with a suboptimal starting gate exit. Supporting this hypothesis, we observe that for the 30 and 60 m races reported in figure 6c, the measured torque at low pedalling rates is higher.

#### (ii) The kilometre 2022 World Championships final: D=1000 m with G=62/13

**Figure 10.**
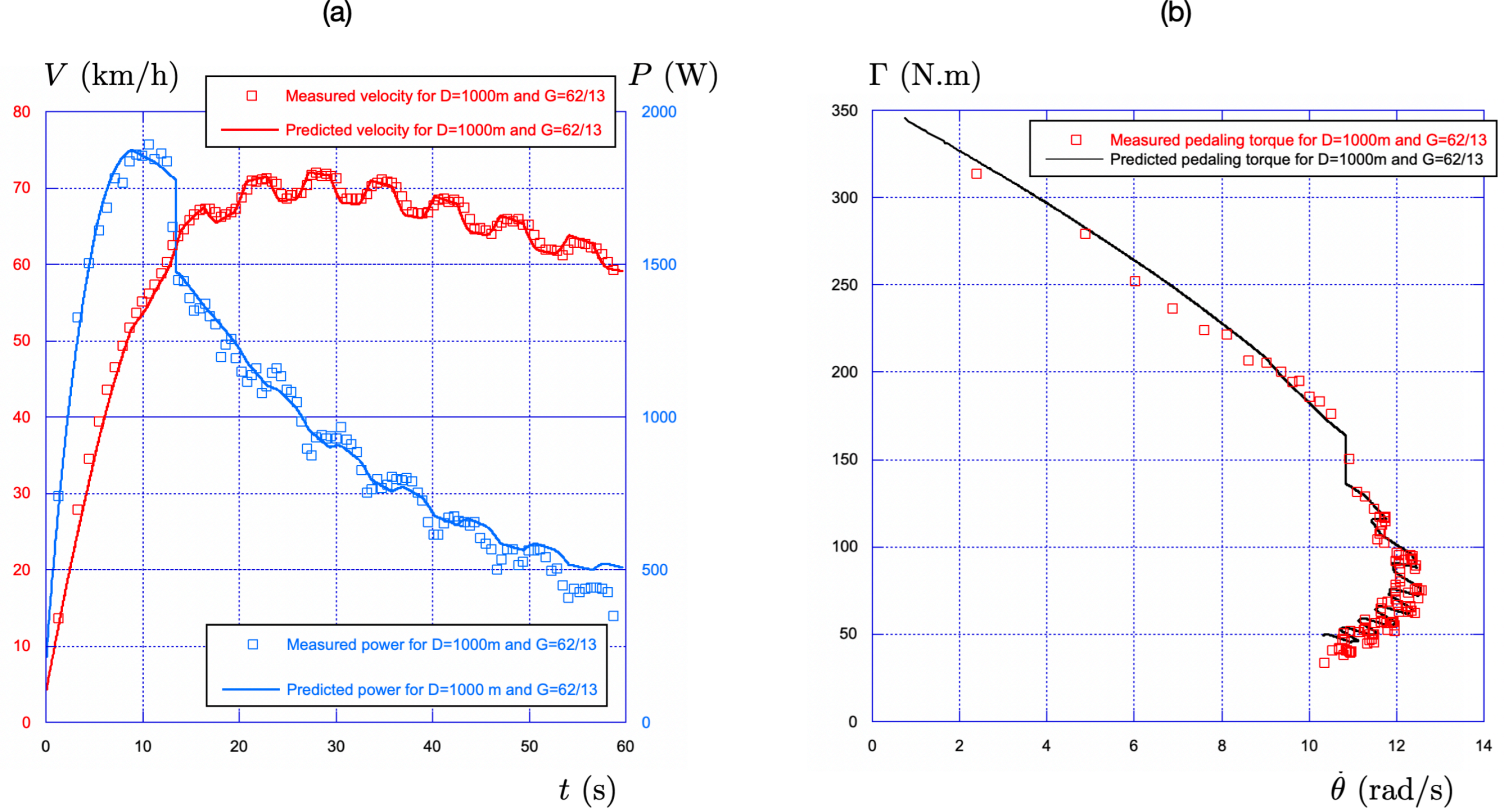
(a) Comparison experiment-theory of the time evolution of the velocity and of the power for the kilometre time trial performed at the 2022 World Championships at SQY with the gear ratio G=62/13. The experimental data are shown with hollow squares (red for velocity and blue for power). The predictions of the effective time theory are shown with solid lines (red for velocity and blue for power). (b) Associated torque-pedalling rate relationship using the same convention: hollow square for experimental data and solid line for the effective time theory.

To test the model over a longer period of time, we present in figure 10 the results obtained with the data measured during the final of the kilometre time trial at the 2022 World Championships held at SQY on Friday 14 November. The air density was measured that day to be $\rho =1.140kg/m^{3}$. The data for the power (in blue) and the velocity (in red), averaged over each pedalling cycle, are presented as a function of time in figure 10a. The power grows and reaches a maximum during the standing phase, which lasts for the first 13 s, then drops by 400 W and finally decreases continuously in the seated phase down to 450 W at the end of the time trial, which the elite sprinter completed in 59.568 s.

Concerning the velocity (in red), it increases during the first 30 s up to 72 km/h and then decreases down to 60 km/h at the end of the race. Eight oscillations are observed, corresponding to the eight turns. All these features are captured by the effective time theory with an accuracy of the order of 1%.

The corresponding torque-pedalling rate relationship is shown in figure 10b. The agreement between the on-field data and the effective time theory is fair for the whole race. As for the 250 m race presented in the previous section, we observe a good description of the jump associated with the stand-sit transition and a fair description of the fatigue during the seated position.

### (c) Back to the impact of the gear ratio, G

Using data recorded during training sessions either to improve strength or endurance, we have shown in figure 7, the impact of the gear ratio G on the final time T for different distances of races ranging from 30 m to 1000 m.

Keeping all parameters the same in the numerical integration of the effective time theory, we measure the evolution of the final time of (numerical) races changing the gear ratio G from 0.5 to 7.

**Figure 11.**
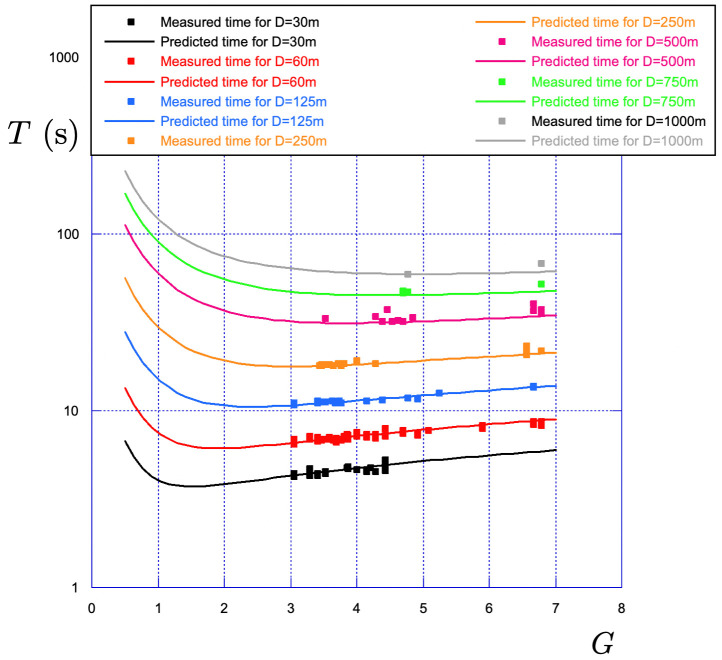
Comparison between the experimental data (squares) and the effective time theory (solid line) for the evolution of the time of the race T with the gear ratio *G* for different distance of races ranging from 30 to 1000 m.

The comparison with the experimental data is shown in figure 11: the first observation is that the agreement between the model and the experimental data is fair for all distances. The second observation is that the model reveals the existence of an optimal gear ratio $G^{⋆}$, which leads to a minimum racing time $T^{⋆}$. This optimal gear ratio increases with the distance of the race.

For D=1000 m, the optimal gear ratio is found to be G=4.84=63/13, which can be compared to the gear ratio G=62/13 used by the elite sprinter during the kilometre time trial of the 2022 World Championships.

## 5. Conclusion

This study introduces a novel theoretical framework, the effective time theory, to model the power output rise and decay of track cycling sprinters. Validated through both ergometer and *in situ* velodrome data, our approach provides a predictive model that closely aligns with experimental observations, offering new insights into fatigue dynamics.

While our findings are directly applicable to standing-start sprint events, further investigations are needed to extend the model to other race formats in track cycling. In particular, races such as the flying 200 m, where gravitational potential energy plays a key role, and team pursuit, which includes recovery phases, represent natural next steps for refining our approach. A better understanding of power distribution in all these contexts could optimize gear selection, pedalling strategies and pacing tactics, leading to improved athlete performance.

Beyond track cycling, our findings have potential implications for a wide range of high-intensity sports where force–velocity–endurance dynamics play a crucial role. The concept of an effective time that controls the maximal power could be particularly relevant to sprint disciplines such as speed skating, sprint kayaking, sprint swimming and even explosive track-and-field events. These sports share common biomechanical constraints, where an initial peak in power output is followed by an inevitable decline due to fatigue.

## Data Availability

All data available from Dryad [34].

## Footnotes

^1^
TTL means transistor–transistor logic: Logic LOW (0): voltage level between 0 V and 0.8 V – Logic HIGH (1): voltage level between 2 V and 5 V.

^2^
This study focuses on sprint efforts performed in the absence of a recovery phase. The applicability of the model to efforts that include recovery therefore remains to be quantified.
